# TG/HDL-C Ratio Is a Risk Factor Associated with CKD: Use in Assessing the Risk of Progression of CKD

**DOI:** 10.3390/pathophysiology29030029

**Published:** 2022-07-17

**Authors:** Ha Hong Nguyen, Ha Hai Tran, Le Thi Nguyen, Thang Nguyen, Nhut Anh Nguyen, Mai Tuyet Vi, Kien Trung Nguyen

**Affiliations:** 1 Department of Physiology, Can Tho University of Medicine and Pharmacy, Can Tho City 900000, Vietnam; nhha@ctump.edu.vn; 2 Faculty of Medicine, Can Tho University of Medicine and Pharmacy, Can Tho City 900000, Vietnam; tranhaiha.haiha23@gmail.com; 3 Department of Physiology, University of Medicine and Pharmacy at Ho Chi Minh City, Ho Chi Minh City 700000, Vietnam; bs.nguyenthile@gmail.com; 4 Department of Pharmacology and Clinical Pharmacy, Can Tho University of Medicine and Pharmacy, Can Tho City 900000, Vietnam; nthang@ctump.edu.vn (T.N.); maivivi127@gmail.com (M.T.V.); 5 Faculty of Pharmacy, Nam Can Tho University, Can Tho City 900000, Vietnam; nanhut@nctu.edu.vn

**Keywords:** TG/HDL-C ratio, chronic kidney disease, eGFR, ACR, Vietnam

## Abstract

Background: Dyslipidemia is highly prevalent in patients with chronic kidney disease (CKD), and the relationship between dyslipidemia and renal function in these patients remains controversial. Our objectives were to determine the triglycerides/HDL-cholesterol ratio (TG/HDL-C), evaluate the correlation between TG/HDL-C and the urine albumin/creatinine ratio (ACR), and estimate the glomerular filtration rate (eGFR) according to MDRD in CKD patients. Methods: A descriptive cross-sectional study was conducted on 152 patients with CKD at the Endocrine Clinic, the University of Medicine and Pharmacy Hospital, Ho Chi Minh City, Vietnam. Study subjects were medically examined and recorded information on the data collection form. Subjects were tested for total cholesterol, triglycerides, HDL-C, LDL-C, urea, creatinine and albumin, urine creatinine, and eGFR according to the MDRD formula. Data were analyzed using SPSS Statistics version 20.0. Results: The average age was 58.08 ± 15.69 years, and the overweight and obesity rate was 54%. Most patients had comorbidities, among which the most common diseases were hypertension and diabetes mellitus. Among the subjects, 57.3% were CKD stage 3 patients, and ACR was in the range of 30–300 mg/g. According to the classification of CKD using GFR and ACR categories, 40.8% of patients were at very high risk. The average TG/HDL-C ratio was 5.09 ± 4.26. There was a medium negative correlation between TG/HDL-C and eGFR (R = 0.44, *p* < 0.01) and a weak positive correlation between TG/HDL-C and ACR (R = 0.34, *p* < 0.01). Conclusions: The TG/HDL-C ratio was a risk factor associated with CKD and was noticeable in monitoring and assessing the risk of progression of CKD.

## 1. Introduction

Chronic kidney disease (CKD) is a global health problem associated with high mortality; the principal outcome of CKD is a progressive loss of kidney function, leading to end-stage renal disease (ESRD) [1,2,3]. Therefore, identifying and managing risk factors associated with mild-to-severe stages of CKD is the best strategy to prevent and delay the progressive outcome of ESRD [4]. Several studies have suggested a vicious circle between loss of renal function and dyslipidemia in CKD, contributing to premature death from cardiovascular disease and other causes [5,6]. Abnormal lipoprotein metabolism has been implicated as a possible cause of CKD [7,8]. CKD was associated with reduced HDL cholesterol levels and elevated triglycerides [8,9]. Many authors have observed the TG/HDL-C ratio [10,11,12,13,14,15].

The TG/HDL-C ratio was found to have a more robust predictive value for cardiovascular events than TG, LDL-C, or the cholesterol/HDL-C ratio [10]. Kurella et al. identified that metabolic syndrome, including elevated triglycerides and low HDL-C, was an independent risk factor for developing incident CKD [11]. In a cross-sectional study that included 5503 subjects (≥19 years of age) who participated in the 2005 Korean National Health and Nutrition Examination Survey in Korean, Hee-Taik Kang et al. found that the TG/HDL-C ratio was independently associated with increased prevalence of CKD [12]. When comparing lipid-related ratios to predict CKD stage 3 or higher in Korean adults (2012), Ji-Young Kim et al. also found that the TG/HDL-C ratio was the only lipid-related ratio independently associated with CKD stage 3 or higher in both sexes among Koreans [13]. A high TG/HDL-C ratio can be associated with chronic renal dysfunction, especially in patients with hypertension and diabetes [14,15]. In 2014, Tsuruya K et al. observed that CKD prevalence, low eGFR, and proteinuria significantly increased with elevated TG/HDL-C in a large Japanese population. With respect to the relationship between dyslipidemia and renal outcome, the authors of one study disclaimed this association in non-diabetic patients with stage 3 to 4 CKD [16], and a large, randomized control trial (2006) showed that statin treatment lowered low-density lipoprotein (LDL) cholesterol but had no substantial effect on kidney disease progression in patients with CKD [17]. Thus, the relationship between dyslipidemia and renal function in CKD patients remains controversial [12,13,14,15,16,17]. Few studies have been conducted on the relationship between the TG/HDL-C ratio and CKD in the Vietnamese population. Therefore, we conducted a study to evaluate the relationship between blood lipoproteins, the ratio of TG/HDL-C, and kidney function in Vietnamese patients with CKD.

## 2. Materials and Methods

### 2.1. Setting and Study Population

A descriptive cross-sectional study was conducted at the Endocrine Clinic, University of Medicine and Pharmacy Hospital, Ho Chi Minh City, from November 2016 to July 2017. We enrolled patients older than 18 years diagnosed with CKD according to the 2012 Kidney Disease Improving Global Outcomes (KDIGO) categories. The exclusion criteria for this study were: patients who (1) had diseases that could affect the results of eGFR or ACR in the urine, such as malnutrition, amputation, muscular dystrophy, muscle atrophy, pregnancy, urinary tract infections, and urinary tumors; and (2) were unwilling to participate in the study. The sample size was estimated by using the formula for a single proportion with an estimated proportion of CKD patients, which was 0.031, as reported in a prior study [18]; an assumed margin of error of 3%; and a confidence level of 95%, resulting in 129 patients. However, the actual sample size that we obtained was 152 CKD patients.

### 2.2. Data Collection and Definitions

KDIGO is defined CKD as kidney damage (with or without decreased GFR) with one or more of the following signs: albuminuria (albumin excretion rate ≥30 mg/24 h, ACR ≥ 30 mg/g or ≥ 3 mg/mmol), urine sediment abnormalities, electrolyte and other abnormalities due to tubular disorders, abnormalities detected by histology, structural abnormalities detected by imaging and history of kidney transplantation, or GFR below 60 mL/min/1.73 m^2^ (with or without kidney damage) for ≥3 months [19,20]. 

Data on eligible CKD patients were extracted from data collection forms, patient medical records, and paraclinical test results. All tests for total cholesterol, triglycerides, HDL-C, LDL-C, urea, blood creatinine, albuminuria, and urine creatinine were performed at the hospital laboratory of the University of Medicine and Pharmacy Hospital according to the standard process. First, patients were asked not to consume anything but water for 12 h before the test and not to eat breakfast on the day of the test; then, 2 mL of blood was and about 10 mL of urine were taken from each patient for testing. Serum urea, serum creatinine, total cholesterol, triglycerides, HDL-C, and LDL-C were quantified by enzymatic colorimetric method, whereby substances participate in color reactions with reagents to form complexes; then the absorbance of the complexes is at the appropriate wavelength [21]. Urine creatinine, urine urea, albumin, and urine protein were quantified by photometric method, whereby substances react with reagents to form colored complexes. The change in optical density measured at the appropriate wavelength was proportional to the concentration of the substance to be quantified in the sample [21].

The following patient characteristics were collected: age, gender, height, weight, BMI, and medical history (CKD, glomerulonephritis, diabetes, nephrotic syndrome, hypertension, systemic lupus erythematosus, and others). Clinical symptoms included edema, pale mucous membranes, hematuria, and others. Paraclinical characteristics included blood lipids (total cholesterol, triglycerides, HDL-C, LDL-C, and TG/HDL-C ratio) and parameters to evaluate kidney function (urea, albumin, creatinine, ACR, and eGFR according to MDRD) [19]. Classification of stages, prognosis assessment, and CKD progression were based on GFR and ACR.

### 2.3. Data Analysis 

Data were analyzed using SPSS Statistics version 20.0. Difference were considered statistically significant when *p* < 0.05. Qualitative variables were described by frequency and percentage, and quantitative variables were presented as mean ± standard deviation. The relationship between two qualitative variables was compared by testing (Chi-squared); between two quantitative variables by *t*-test with 95% confidence. The correlation coefficients of two quantitative variables were determined by the Pearson correlation coefficient if the variables were normally distributed and by the Spearman correlation coefficient if the variables non-normally distributed. The convention proposed by Guilford (1965) was used to interpret the correlation coefficients of the sample (Table 1).

### 2.4. Ethics Approval

The council of Ho Chi Minh University of Medicine and Pharmacy approved our study (No. 91/ĐHYD-HĐĐĐ, dated 30 March 2017). All study participants gave their consent and agreed that their personal information would be kept private. This study did not harmed the patients’ health.

## 3. Results

### 3.1. Patient Characteristics

Among 152 patients, 55% were men; the mean age was 58.08 ± 15.69. The average BMI was 23.45 ± 3.07 kg/m^2^, and the overweight/obesity rate accounted for 54% of subjects. The main clinical symptom was pale mucous membranes; less common symptoms included edema and hematuria. Most patients had comorbidities, the most common of which were hypertension (63%) and diabetes (24%). The group of patients with TG/HDL-C < 4 accounted for 53.3% of subjects—higher than that of TG/HDL-C ≥ 4. A proportion of 57% of subjects were CKD stage 3 patients, and the urine ACR was in the range of 30–300 mg/g. According to the classification of CKD using GFR and ACR categories, 41% of patients were at very high risk (Table 2).

### 3.2. Characteristics of Patients with TG/HDL-C < 4 and ≥ 4

The difference was statistically significant in BMI, triglycerides, HDL-C, eGFR, and ACR between two groups of patients with TG/HDL-C < 4 and ≥ 4 (*p* < 0.05). TG/HDL-C < 4 occurred most commonly among patients at moderate risk of kidney disease; TG/HDL-C ≥ 4 was primarily associated with the very high-risk group (*p* < 0.05). Patients with TG/HDL-C < 4 mostly had stages 1 and 2 CKD; in contrast, TG/HDL-C ≥ 4 was more common in patients with stages 3, 4, and 5 CKD (*p* < 0.05) (Table 3).

### 3.3. Correlation of Blood Lipids and TG/HDL-C with eGFR and ACR

There was a medium negative correlation between TG/HDL-C and eGFR (R = 0.44, *p* < 0.01) and a weak positive correlation between TG/HDL-C and ACR (R = 0.34, *p* < 0.01) (Table 4).

## 4. Discussion

### 4.1. Patient Characteristics

Among 152 patients with CKD, 55% were men, and 45% were women—similar to the sample in a study by Chih-I Ho et al., which included 56.1% men and 43.9% women [22]. Therefore, the risk of CKD was similar between the sexes. The average age of subjects in our study was 58.08 ± 15.69 years old, compared to 63.8 years old in a study by Kazuhiko Tsuruya [14]. According to annual statistical data on chronic kidney disease in the United States, the average age of CKD patients is 52.3 years old [15]. Previous histopathological studies suggested that age is associated with glomerular fibrosis. Age was identified as a risk factor for CKD progression, and the proportion of loss of renal function was higher in older patients than in younger patients [23]. Based on the BMI classification of the Western Pacific Regional Office, the overweight and obesity rate among subject in this study was 54%, indicating that the increase in the overweight and obesity rate was a significant contributor to CKD progression [15]. An overview of kidney disease facts and statistics for the United States, including CKD, indicated that older age and BMI ≥ 30 kg/m^2^ are associated with CKD [15]. 

Clinical symptoms of CKD included hypertension, pale mucous membranes, edema, and hematuria, among which pale mucous membranes and hypertension conditions accounted for a high rate, as patients often had chronic anemia. Most patients had comorbidities, of which the common diseases were hypertension (63%) and diabetes mellitus (24%). Over time, high blood pressure could damage blood vessels throughout the body, reducing the blood supply to vital organs, such as the kidneys, damaging the kidney’s tiny filtering units. As a result, the kidneys might stop removing waste and extra fluid from the blood; therefore, hypertension is a significant cause of kidney failure, especially CKD. Long-term diabetes also causes atherosclerosis of large blood vessels, including renal arteries, narrowing blood vessels and resulting in high blood pressure and kidney failure. In this study, the mean concentration of total cholesterol, triglycerides, HDL-C, and LDL-C was 192.80 ± 58.42 mg/dL, 208.33 ± 140.92 mg/dL, 47.22 ± 14.94 mg/dL, and 121.0 ± 44.45 mg/dL, respectively. Recent guidelines indicated that triglycerides and HDL-C should be routinely evaluated in patients with type 2 diabetes [24]. A high TG/HDL-C percentage was significantly associated with CKD risk, independent of hypertension, diabetes, and obesity. However, high TG/HDL-C levels were related to chronic kidney disease, especially in patients with hypertension and diabetes [24]. TG/HDL-C has been identified as an indicator of insulin resistance and atherosclerosis, in addition to their comorbidities [13]. Increased TG/HDL-C may be associated with glomerulosclerosis, which is related to the development of CKD. Although its mechanism of development is not clearly understood, CKD shares many risk factors with cardiovascular disease (CVD). In addition to the traditional risk factors of CKD, including hypertension, diabetes mellitus, and aging, chronic inflammation, oxidative stress, and obesity are indicators of renal dysfunction development [13]. On a cellular level, it has been shown that when mesangial cells are stimulated via exposure to lipids, they secrete proinflammatory cytokines, such as interleukin-6, tumor necrosis factor-α, and transforming growth factor-β. Glomerular mesangial cells and vascular smooth muscle cells have molecular similarities, pointing to a common underlying pathophysiology in the development of atherosclerosis and glomerulosclerosis [13]. Insulin resistance mediates diabetes, obesity, hypertension, lipid abnormalities, and atherosclerosis, all risk factors for CKD and CVD. Insulin resistance is also a significant risk factor for the progression of renal dysfunction in nondiabetic subjects. TG/HDL-C is an indicator of insulin resistance and cardiovascular mortality. Increased levels of small and dense LDL-C particles, which are highly atherogenic, are associated with elevated TG/HDL-C [13]. These results show that increased TG/HDL-C may lead to glomerulosclerosis and CKD. Compared with other lipid-related ratios investigated in this study, the stronger relationship between CKD and TG/HDL-C may be due to most patients having comorbid hypertension or diabetes. Furthermore, the average TG/HDL-C ratio of subjects in our study was 5.09 ± 4.26—higher than the results reported in a study by Chih-I Ho (2.3 ± 2.6) [22]. This could be because our study was conducted with a small sample size of patients with CKD, whereas Chih-I Ho’s study was performed on a large population to screen health check-ups and CKD surveys.

In addition, we found that the average eGFR was 52.50 ± 20.10 mL/min/1.73 m^2^, of which stage 3 of CKD was the most common (57%). This was similar to the results of a study by Le Quoc Tuan and Dang Huynh Anh Thu (2017), who recorded the rate of stages 1-5 CKD as 18%, 30%, 36%, 10%, and 6%, respectively [25]. The urine ACR was within the range of 30–300 mg/g (41%) in this study. Microscopic hematuria was one of the symptoms of glomerular damage. According to an annual report on CKD in the United States, the percentage of ACR > 10 mg/g was 32%, whereas the rates of ACR 30–300 mg/g and ACR > 300 mg/g were 8.5% and 1.4%, respectively. With respect to evaluation of the overall distribution of eGFR and ACR, the report also showed that elevated ACR was associated with reduced renal function [15]. Similarly, according to the classification of CKD using GFR and ACR categories in this study, up to 41% of patients were at very high risk.

### 4.2. Characteristics of Patients with TG/HDL-C < 4 and ≥ 4

When considering two groups of patients with TG/HDL-C ratios < 4 and ≥ 4, we found a significant difference in BMI, triglycerides, HDL-C, eGFR, and ACR (*p* < 0.05). TG/HDL-C ≥ 4 was found primarily in the very high-risk group (*p* < 0.05) and was more common in patients with stage 3 and higher CKD (*p* < 0.05). Research by Chih-I Ho et al. (2015) showed increases from the lowest to the highest quartile of the TG/HDL-C ratio. Men and women in the highest TG/HDL-C ratio quartile (>2.76) had a 1.4-fold and 1.74-fold greater risk of CKD than those in the lowest quartile (≤1.04), respectively, independent of confounding factors. Therefore, A TG/HDL-C ratio ≥2.76 may be helpful in clinical practice to detect subjects with worsened cardiometabolic profiles who require monitoring to prevent CKD [22].

### 4.3. Correlation of Blood Lipids and TG/HDL-C with eGFR and ACR

There was a medium negative correlation between TG/HDL-C and eGFR (R = −0.44, *p* < 0.01). Dyslipidemia might decrease the glomerular filtration rate and accelerate the rate of progression of kidney disease by several mechanisms [7]. First, phospholipid and cholesterol reabsorption in renal tubular epithelial cells released inflammatory factors and tissue damage [22]. Second, lipoproteins stimulated the formation of inflammatory cytokines and induced renal atherosclerosis [26]. The loss of renal function facilitated hypertriglyceridemia and accelerated dyslipidemia, resulting in a vicious cycle between dyslipidemia and decreased renal function [22,26]. Moreover, the research results of Kazuhiko Tsuruya not only emphasized the vital role of Triglyceride/HDL-C as a factor related to CKD progression, especially in patients with diabetes and hypertension, but also noted the benefit of positive management of dyslipidemia in preventing the morbidity and progression of renal failure [14]. This research showed a weak positive correlation between TG/HDL-C and ACR (R = 0.34, *p* < 0.01). Many studies have demonstrated that a high TG/HDL-C ratio is a valuable marker for proteinuria abnormalities [27,28,29]. Abnormalities in lipid metabolism are believed to contribute to the progression of kidney disease [30,31].

This study is subject to some limitations. First, the observational nature of the cross-sectional research reduced the reliability of establishing a cause-and-effect relationship. Second, the short study period and small sample size might not be sufficient to comprehensively assess the impact of dyslipidemia on renal function. Third, some potential risk factors were not analyzed, such as the history of lipid-lowering drugs and other comorbidities. Therefore, we recommended that further studies be conducted with larger sample sizes in patients with annual physical examinations and simultaneously consider the potential risk factors mentioned above to evaluate the role of TG/HDL-C for CKD in the general population. Otherwise, the MDRD and CKD-EPI equations are the most popular methods for estimating GFR in patients aged 18 years and older. Both of these methods may allow us to observe that CKD is present despite a serum creatinine concentration that appears to fall within or just above the normal reference interval; thus, future studies should determine GFR by CKD-EPI equation and directly compare the impact of these equations on the research results.

This study is one of the few studies in Vietnam and globally to show the clinical significance of the ratio of TG/HDL-C for assessment of kidney function progression in Vietnamese patients with CKD. This helped to monitor and evaluate the risk of developing CKD more effectively. In addition, this result also emphasizes the importance of the continued need for medical interventions to treat dyslipidemia in CKD patients.

## 5. Conclusions

The TG/HDL-C ratio was found to be a risk factor associated with CKD and should be used in early screening, monitoring, and assessment of the risk of CKD progression. During annual health check-ups, if an increase in TG/HDL-C is observed, doctors should to monitor the patient’s kidney function.

## Figures and Tables

**Table 1:** Guilford’s interpretation of the magnitude of significant correlations (1956).

|R|	Interpretation
<0.20	Almost no relationship
0.20–0.40	Low correlation
0.40–0.70	Moderate correlation
0.70–0.90	High correlation
>0.90	Very high correlation

**Table 2:** Patient characteristics.

General Characteristics	Frequency	Percentage (%)
Average age	58.08 ± 15.69 years	
BMI	23.45 ± 3.07 kg/m^2^	
Underweight	8	5
Normal	62	41
Overweight/obesity	82	54
Gender		
Male	84	55
Female	68	45
Clinical symptoms		
Edema		
Yes	32	21
No	120	79
Pale mucous membranes		
Yes	81	53
No	71	47
Hematuria		
Yes	17	11
No	135	89
Other symptoms		
Yes	8	5
No	144	95
Medical history		
Glomerulonephritis		
Yes	31	20
No	121	80
Diabetes		
Yes	36	24
No	116	76
Nephrotic syndrome		
Yes	12	8
No	140	92
Hypertension		
Yes	96	63
No	56	89
Systemic lupus erythematosus		
Yes	7	5
No	145	95
Blood lipids (mg/dL)		
Cholesterol	192.80 ± 58.42	
Triglycerides	208.33 ± 140.92	
HDL-C	47.22 ± 14.94	
LDL-C	121.0 ± 44.45	
TG/HDL-C	5.09 ± 4.26	
TG/HDL-C < 4	81	53
TG/HDL-C ≥ 4	71	47
Parameters to evaluate kidney function		
Urea (mg/dL)	49.59 ± 25.81	
Creatinine (mg/dL)	1.50 ± 0.80	
eGFR (mL/min/1.73 m^2^)	52.50 ± 20.10	
ACR (mg/g)	605.25 ± 1358.07	
<30 mg/g	33	22
30–300 mg/g	62	41
>300 mg/g	57	37
Stages of CKD		
Stages 1 and 2	45	30
Stage 3	87	57
Stage 4	16	10
Stage 5	4	3
Classification of CKD based on GFR and ACR		
Low risk	0	0
Moderate risk	53	35
High risk	37	24
Very high risk	62	41

**Table 3:** Characteristics of patients with TG/HDL-C < 4 and ≥ 4.

Characteristic	TG/HDL-C		*p*
	<4 (*n* = 81)	≥4 (*n* = 71)	
	*n* (%)	*n* (%)	
Average age (mean ± SD)	57.85 ± 17.46	58.34 ± 13.50	0.85
BMI (mean ± SD)	22.91 ± 3.26	24.07 ± 2.73	0.02
Blood lipids (mg/dL)			
Cholesterol	193.33 ± 57.18	192.18 ± 60.21	0.90
Triglycerides	123.60 ± 43.10	304.99 ± 151.44	<0.01
HDL-C	54.48 ± 15.58	38.94 ± 8.60	<0.01
LDL-C	120.80 ± 44.91	121.23 ± 44.23	0.95
Parameters to evaluate kidney function			
eGFR (ml/min/1.73 m^2^)	59.38 ± 19.33	44.65 ± 20.16	<0.01
ACR (mg/g)	397.50 ± 103.30	842.27 ± 201.54	0.04
Stage of CKD			
Stages 1 and 2	34 (42%)	14 (20%)	0.003
Stages 3, 4, and 5	47 (58%)	57 (80%)	
Classification of CKD based on GFR and ACR			
Moderate risk	34 (42%)	19 (27%)	<0.05
High risk	23 (28.4%)	14 (20%)	
Very high risk	24 (29.6%)	38 (53%)	

**Table 4:** Correlation of blood lipids and TG/HDL-C with eGFR and ACR.

Blood Lipids	eGFR		ACR	
	Correlation	*p*	Correlation	*p*
Cholesterol	R = −0.14	0.09	R = 0.22	<0.01
Triglycerides	R = 0.20	0.02	R = 0.38	<0.01
HDL-C	R = −0.23	<0.01	R = −0.05	0.55
LDL-C	R = −0.13	0.12	R = 0.21	<0.05
TG/HDL-C	R = −0.44	<0.01	R = 0.34	<0.01

## Data Availability

Not applicable.
